# The effects of climate change-induced flooding on harvest failure in Burkina Faso: case study

**DOI:** 10.3389/fpubh.2023.1166913

**Published:** 2023-08-08

**Authors:** Charlotte Müller, Windpanga Aristide Ouédraogo, Maximilian Schwarz, Sandra Barteit, Rainer Sauerborn

**Affiliations:** ^1^Heidelberg Institute of Global Health (HIGH), Faculty of Medicine and University Hospital, Heidelberg University, Heidelberg, Germany; ^2^Faculty of Health Science and Medicine, University of Lucerne, Lucerne, Switzerland; ^3^Centre de Recherche en Santé de Nouna, Nouna, Burkina Faso; ^4^Remote Sensing Solutions GmbH, Munich, Germany

**Keywords:** sub-Saharan Africa, extreme weather events, low-income country, agriculture, subsistence-farming, extreme rainfall, climate change, flooding

## Abstract

**Background:**

Climate change leads to more frequent and severe extreme weather events including floods, heatwaves, heavy rainfalls, and droughts. In contrast to the majority of research on weather extremes in sub-Saharan Africa, which focus primarily on how a lack of rainfall causes droughts, this paper aims to elucidate the effect of flooding on harvest failure in rural Burkina Faso.

**Methods:**

We conducted a case study in north-western Nouna, Burkina Faso, between August and December 2021 covering a study population of *n* = 180 participants. The study comprised four components: (i) interviews with farmers (*n* = 180) on whether any of their fields had been inundated and if so, on harvest loss on these fields; (ii) determining the feasibility of using Sentinel-2 satellite images to validate study participants reports of floods; (iii) characterizing short-term weather including frequency and duration, of extreme rainfall events within the study area, as well as comparing cumulative rainfall (long-term) over the past 50 years; and (v), estimating both the food energy and economic loss of harvest failure due to flooding.

**Results:**

49% of interviewed farmers (*n* = 88) reported that floods had damaged at least one of their fields. Some fields (*n* = 13, 7%) had no harvest due to flooding, while some farmers (*n* = 14, 8%) had lost part of their harvest. Images from the Sentinel-2-Satellite indicated that reported and remotely observed flooding were consistent. According to time series of data from the local weather station, there has been an increase irregular rainfall distribution and at the same time of cumulative annual rainfall in Nouna. Furthermore, a first illustrative calculation allowed us to estimate the amount of energy lost when one hectare of a common crop is flooded.

**Conclusion:**

This case study demonstrated that flood-related harvest failures leading to crop losses in sub-Saharan Africa, exemplified by Burkina Faso, are likely to be substantial. This study serves as a proof-of-principle for flooding effects on food security. This could provide more detail for agricultural adaptation and mitigation strategies. Inundation-vulnerable fields need alternative and novel management practices, which may only be effectively implemented if agricultural institutions and national policy-making bodies receive evidence of flooding e.g., from remote sensing.

## 1. Introduction

### 1.1. Background

Climate change has raised the global mean temperature by 1.21°C in 2021, compared to higher the baseline pre-industrial period of 1850 and 1900 (1). One major effect of climate change is an increase in extreme weather events including heat waves, heavy rainfall, which may lead to flooding and landslides, storms and droughts (2). The global increase in extreme rainfall events may result in frequent and large scale flooding events damaging people, houses, and crops (3). It is not only cumulative rainfall that affects plants, agriculture, livestock, and humans, but also the distribution of rainfall, which throughout the rainy season may include both heavy rainfall events and so called mini-droughts, which are shorter periods of irregular drought. A study by Bellprat et al. (4) found that recent extremes in dry and wet rainy seasons have caused severe socio-economic damage in Mozambique, in Southern Africa. In other African regions, a lack of precipitation may cause severe droughts, which increases the risk of wildfires and harvest losses, all of which adversely impact food security (5).

The health impacts of climate change are well documented (6). Numerous studies identified several key factors relating direct and indirect effects of climate change on human health. In low-resource contexts, such as Burkina Faso, where subsistence farming is common, harvest failure threatens food security and consequently child undernutrition, as households largely eat what they grow (7). A review by Phalkey et al. (8) analyzed 15 studies to determine how different pathways of climate change affect childhood undernutrition in subsistence farming in low- and middle-income countries. Extreme weather events, like droughts, are one of the most frequent and severe global effects of climate change on food security. Rodriguez-Llanes et al. (9) identified climate change as the leading cause of harvest shortages and agricultural variety loss, which threatens food security in rural regions where subsistence farming is dominant. Mank et al. (10) emphasized that climate change predominantly affected the most vulnerable groups and regions which had already been facing food insecurity (10). Kogan et al. (11) reported a 23% undernourishment rate in sub-Saharan Africa in 2017, which is predicted to rise owing to the effects of climate change. Furthermore, they found malnutrition, especially a lack of macro- and micronutrients, as the primary cause of child undernourishment, due to insufficient harvests and a lack of crop diversification (11). In Burkina Faso, where the rural population is particularly reliant on the agricultural sector, there is a paucity of literature on food security in relation to extreme weather exposures, such as flooding (12).

This study aims to provide a basis for better characterizing the magnitude and impact of flooding caused by heavy rainfall on agricultural fields and, consequently, on harvest failure. Specifically, we focus on the following four research questions:

(1) What are farmers' self-reported frequency, extent, and impact of flooding on their harvest?
(2) Can satellite images be used to identify farmers' self-reported extent of flooding (proof of concept)?
(3) How much extreme rainfall occurs throughout the study year (short-term trends), in relation to the cumulative rainfall in the past 50 years (long-term trends)?
(4) How high are the losses of food energy (kcal/ha) and household income? As a result of flooding?

## 2. Study population and methods

This study is reported in accordance with the STROBE (Strengthening the Reporting of Observational studies in Epidemiology) guidelines and was embedded in a larger study on “Climate Change and Health in sub-Saharan Africa” (13).^1^

### 2.1. Study setting and sample size

The study was conducted in the Kossi province of the Nouna HDSS [health and demographic surveillance system (14)], Burkina Faso, between August and December 2021, the period of highest agricultural An HDSS collects demographic data (births, death, in-/out-migration) routinely in a geographically defined contiguous population. Currently, the Nouna HDSS comprises about *n* = 120.000 inhabitants (15).

A simple random sample of 59 villages from the Nouna HDSS was used to select 180 households in 25 villages. Initially, *n* = 210 participants were included. However, as 30 of the study participants were couples owning and working in the same field, only one member of the household was interviewed. Therefore, the study includes 180 participants between the ages of 20 and 65 years.

### 2.2. Study procedures

In the following, we describe in more detail the four study components.

#### 2.2.1. Questionnaire

In December 2021, a total of ten fieldworkers administered the questionnaire via face-to-face interviews. To ensure that the survey is anonymous, every participant was assigned a seven-digit identifier. Field workers were trained to interview farmers using tablets with an electronic questionnaire using Survey Solutions software (16). We elicited information about the magnitude of flooding (frequency during the agricultural season), as well as effects on harvest yields (see Appendix I for the full questionnaire). Specifically, the questionnaire comprised six questions, as follows: (1) the number of fields owned by each farmer and type of food or cash crop sown on each; (2) any experience of flooding on any of these fields between the sowing and harvesting time in 2021. If a study participant answered this question with “No” the survey ended, else, the survey continued with: (3) the number of flooded fields, as well as the type of crop sown on these fields; (4) number of days the fields were flooded. Finally, (5) the perceived flooding effect on harvest (5-Likert-scaled answer options: 1 = no effect to 5 = complete loss of harvest).

#### 2.2.2. Satellite images to validate localization of flooded fields

As a proof-of-concept, we explored the potential of Sentinel-2 satellite imagery to validate the localization of flooded fields within the study area. We utilized the reported time of inundation as well as weather data to identify flooded fields within the study area. The satellite images were downloaded at the time point of the greatest reported inundation, as well as before and after the floods, for comparison. Satellite-based images were sourced from the Copernicus Open Access Hub, provided by the Sentinel-2 satellite of the European Union (17). In a first step, the open-source Quantum Geographic Information System (QGIS) was used to analyse and visualize the obtained satellite and field data. In a second step, the HDSS geographic map was merged with the downloaded satellite image. Using the geographically assigned field numbers of study participants, we were able to indicate their field location on the map.

#### 2.2.3. Weather data to characterize short-term and long-term trends

To characterize rainfall patterns of the past five decades for the region of Nouna, we employed two main data sources: (i) weather data was obtained from the climate data set Climatic Research Unit gridded Time Series (CRU TS) by Harris et al. (18), which includes the variables of temperature, precipitation, vapor pressure, wet days, and cloud cover for all land domains on Earth (except Antarctica); (ii) since 2020, five weather stations in the Nouna study area collected data on precipitation, wind speed, wind direction, solar radiation and temperature and were the source for the short-term characterization of weather, in particular to determine if the frequency of heavy rainfall has increased in comparison to the past (19).

#### 2.2.4. Energy value calculation of crop and cash cereal production

Another component constituted the energy value calculation of food and cash crop production based on the paper by Belesova et al. (20). We estimated the energy value of one hectare of the respective field rather than the value per (in kilocalories) to calculate the number of calories lost from flooding (for details of the calculation, see Appendix II). With this calculation, the maximum potential food energy from one hectare was determined by calculating the weight of the food crop per hectare multiplied by the calorie of the crop type (see Appendix II).

For the second calculation, the energy value of the crop and cash cereal combined, the energy value of the food crop was added to the energy value of the amount of millet, which may be purchased with the profits from the sale of the cash crop. Therefore, we calculated the financial value of the cash crop and how much millet one could buy for this amount of money to consider this in the calculation. With this method the energy value in kcal/ha for crop cereal can be calculated directly and for cash crop indirectly.

(1) Energy value of crop cereal produce in kilocalories/hectare:

$$\begin{array}{l} E_{f}=\underset{i}{\sum }\left(h_{i}\times c_{i}\right)\; \end{array}$$

(2) Energy value of crop and cash cereals in kilocalories/ hectare:

$$\begin{array}{l} E_{fc}=\underset{i}{\sum }\left(h_{i}\times c_{i}\right)\pm c_{i}\times \underset{c}{\sum }\left(h_{c}\times p_{c}\right)÷p_{i} \end{array}$$

Variables:

E Energy value

i food crop: millet, sorghum

c cash crop: cotton, sesame

h weight (kg) of the crop per ha

c caloric value of 1 kg of the food crop

p market price of 1 kg of food and cash crop

## 3. Results

In the study population the average field size was 1.6 ha (min: 0,77 ha; max:3.7 ha) (see Appendix III).

### 3.1. Flooding events

**Figure 1:**
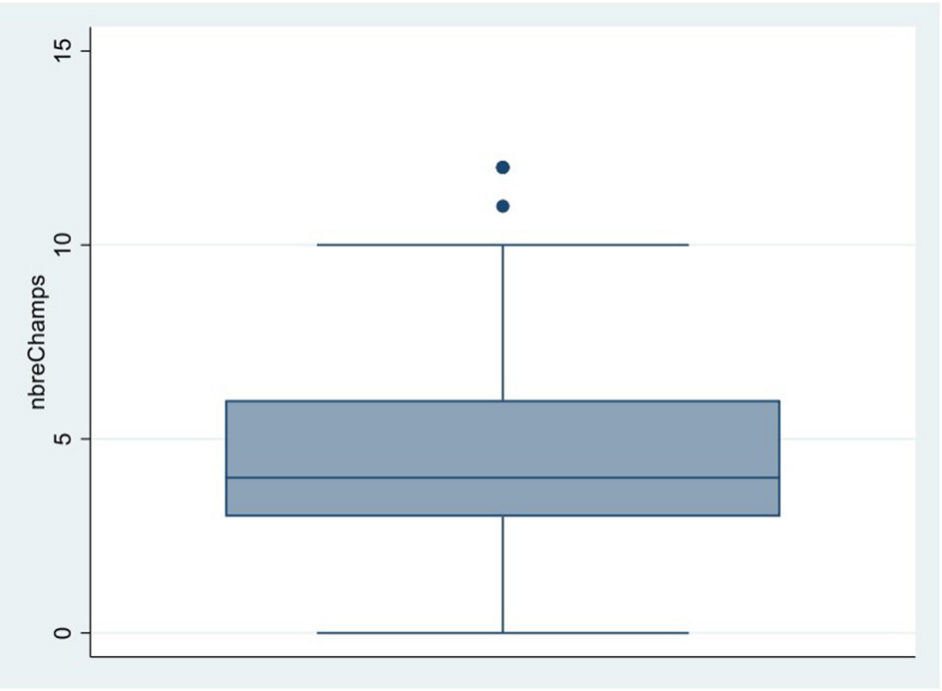
Boxplot of the number and mean of fields from the study population.

All *n* = 180 study participants completed the questionnaire (response rate = 100%). The total number of fields that were included as part of this study was 834, with each farmer managing an average of 4.63 fields (Figure 1). Regarding the question “Was at least one of your fields flooded between the sowing and harvest period?”, *n* = 88 farmers (49%) experienced inundation on one or more fields during the harvest period of 2021, while the remaining *n* = 92 farmers (51%) reported no instances of flooding.

Out of 834 fields (100%), 175 fields (21%) were flooded. Among the 88 households that reported at least one flooded field, 41% (*n* = 36) experienced flooding on a single field, 34% (*n* = 30) on two fields, and one participant had six flooded fields. In total, 80 farmers (44%) reported flood-related harvest loss.

**Figure 2:**
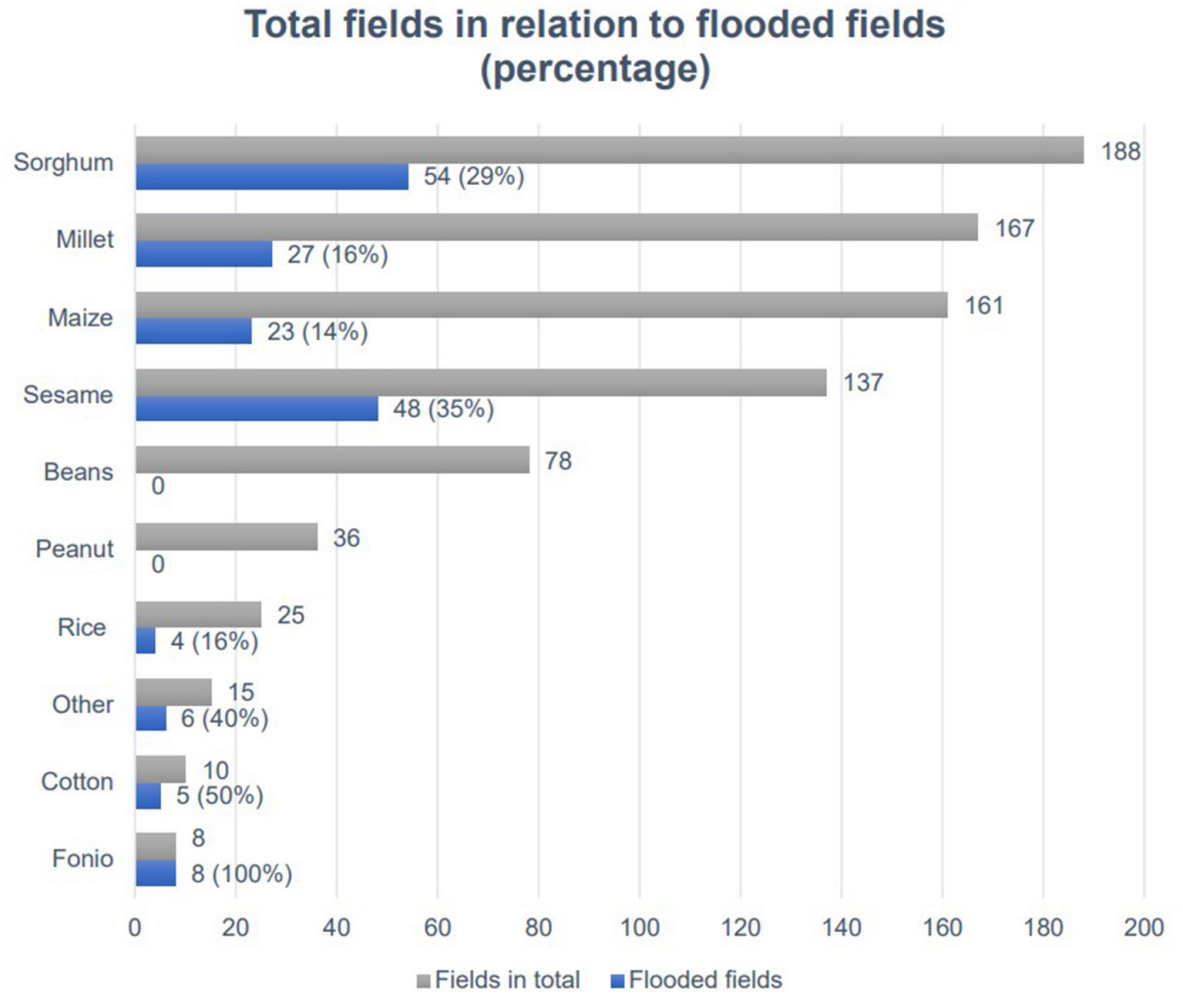
Percentage of all flooded fields out of all fields, by type of crop.

Figure 2 displays the types of crops affected by flooding in relation to the total number of fields. Sorghum was the most affected crop, with 54 inundated fields, followed by sesame with 48 and millet with 27. Flooding impacted eight out of 10 major crop types cultivated in the study area.

**Figure 3:**
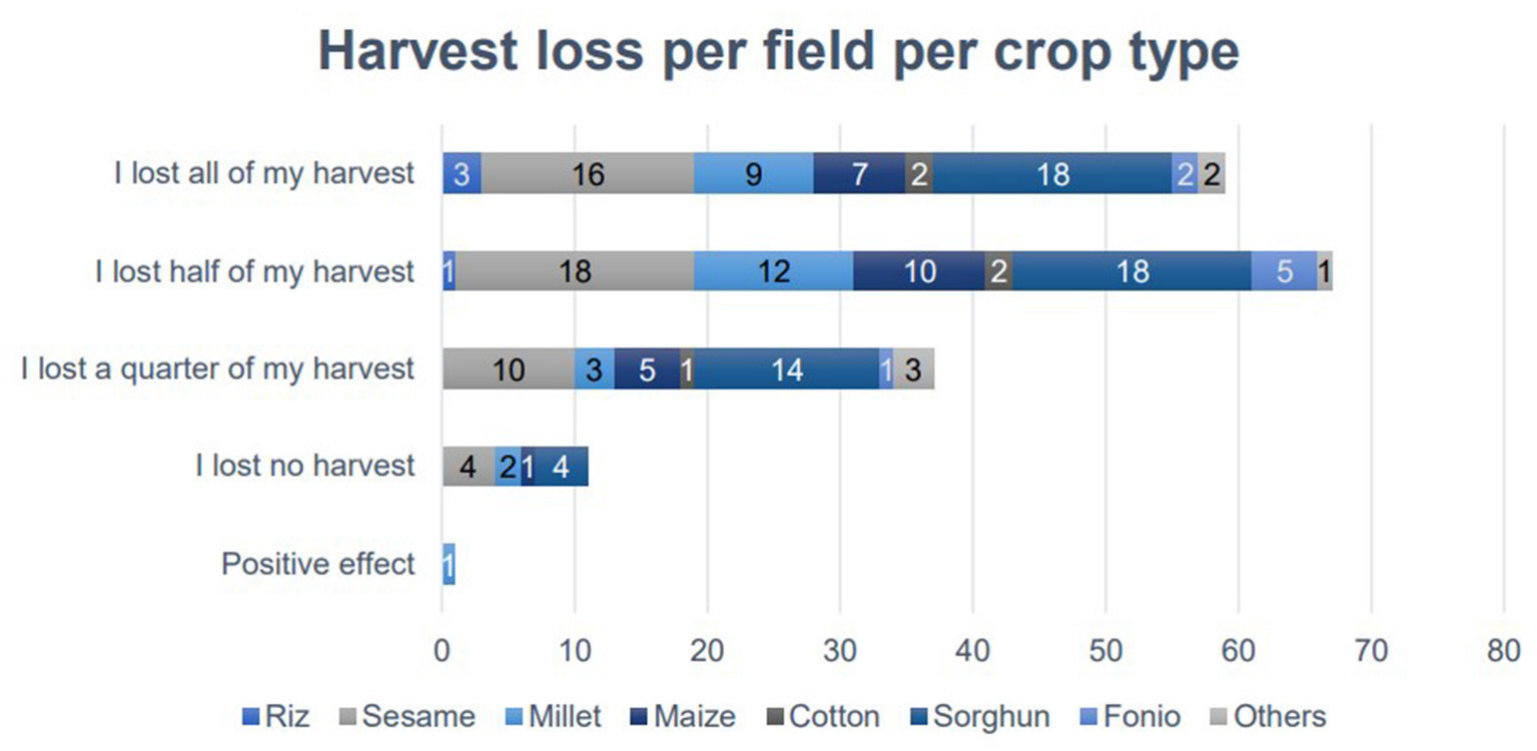
Responses to harvest loss by crop type.

In total, 59 fields (33%) lost all of their harvest. Overall, 33% (*n* = 59) of the flooded fields were cropless. From the whole study population including 834 fields (100%), 7% (*n* = 59) of all fields had no harvest outcome. Figure 3 shows to what extent the inundated fields were affected.

The median duration of flooding was 33 days, with a range from 1 to 90 days. Out of the *n* = 88 participants with inundated fields, seven reported no adverse effects of flooding on their harvest. Consequently, flooding affected the crops of 81 households (45%) of the study population.

**Figure 4:**
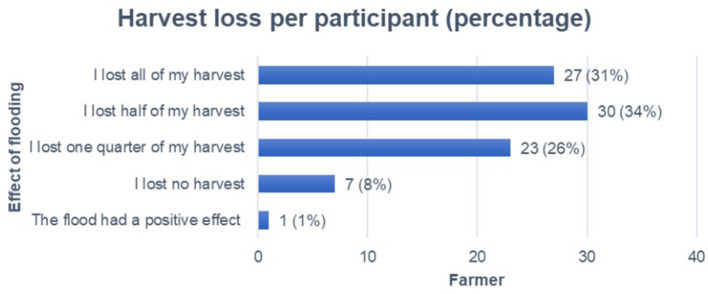
Flooding effect on harvest per farmer, percentage in brackets.

Figure 4 presents the magnitude of harvest loss across five answer categories. A total of *n* = 27 participants reported a complete loss of their harvest due to flooding on at least one field, *n* = 30 reported losing half of their harvest, and seven reported no harvest loss.

Figure 2 displays the relation between the total number of fields and flooded fields for ten distinct categories of crops (sorghum, millet, maize, sesame, beans, peanut, rice, cotton, fonio, and other). All fields that had the crop type fonio planted were inundated, while beans and groundnut fields were not flooded.

Considering the whole study population, the average farmer has about five fields. Of the 180 participants, 88 farmers were affected by flooding, with an average of two inundated fields. Therefore, 40% of their harvests were affected by flooding. By considering the average number of inundated fields, field size and percentage of flooding, we estimated that 158.2 hectares of harvest were destroyed and due to inundation.

### 3.2. Satellite images to visualize evidence of flooding events

**Figure 5:**
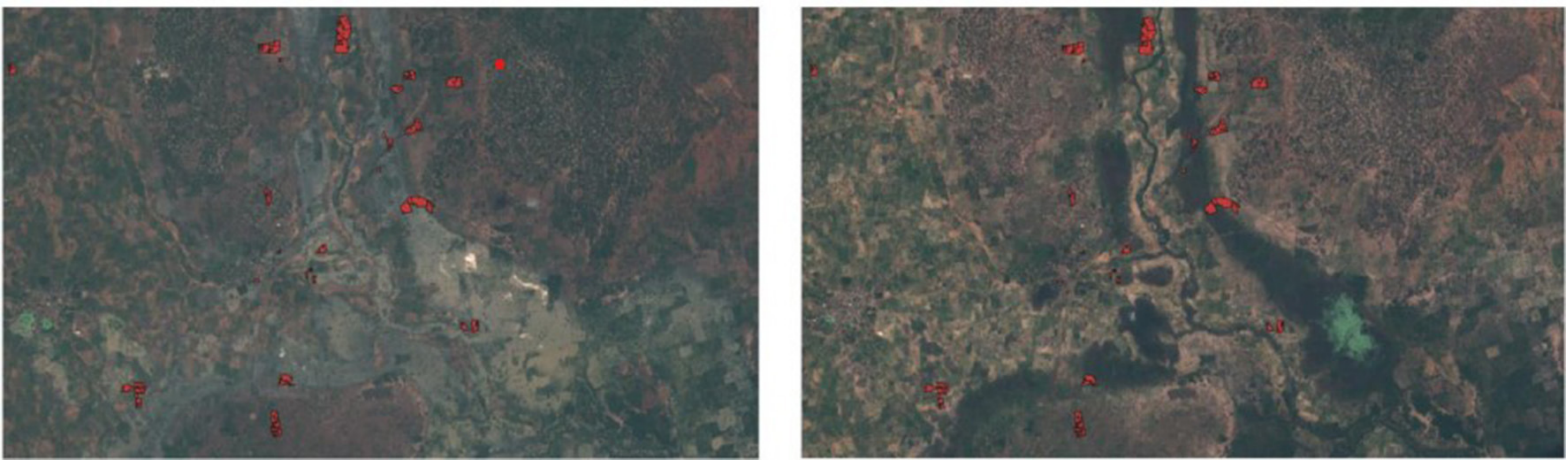
True color images of one part of the HDSS area while flooding (20.08.2021) and after flooding (24.09.2021).

We used satellite images (true color images) of the Sentinel-2 satellite at two different time points at the peak of the rainy seasons (20 August 2021 and 24 September 2021) of the region Boron (between Toni and Tonkoroni) in the Nouna HDSS to be able to visually characterize the flooded areas and visually verify reporting's of study participants regarding flooded areas. Figure 5 shows the scenes with the fields of study participants of this area highlighted in red.

The first satellite image captured on August 20, 2021, showed that considerable rainfall had occurred (from July 25-29, 2021), making August (typically) the wettest month of the year. These heavy rains caused numerous flooded areas. Furthermore, they caused river flooding, obscuring its course. The second image taken on the September 24, 2021 visualizes the landscape after the flooding.

**Figure 6:**
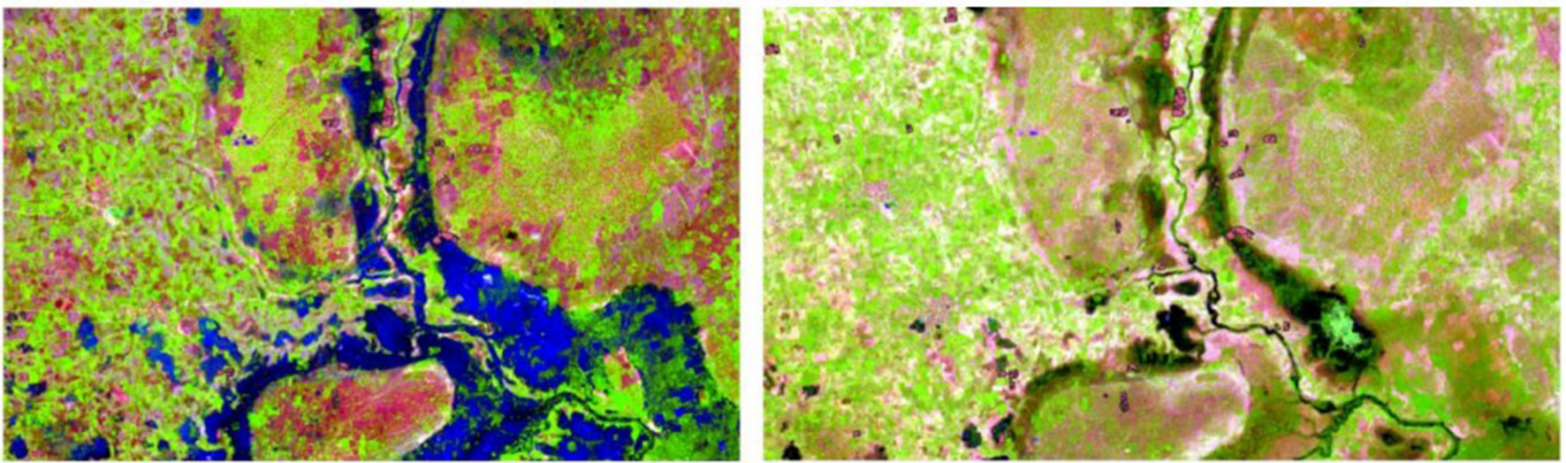
Adapted color images of the same part of the HDSS area while flooding (20.08.2021) and after flooding (24.09.2021).

In Figure 5, the difference between the flooding and inundated areas can be seen in real tone color. Figure 6 shows a different band combination (Color representation: R: SWIR, G: NIR, B: Red.), which enables for a better visualization of water based on the spectral properties of water. In this composite, water is visualized in bright blue.

### 3.3. Weather data to characterize short-term and long-term trends

**Figure 7:**
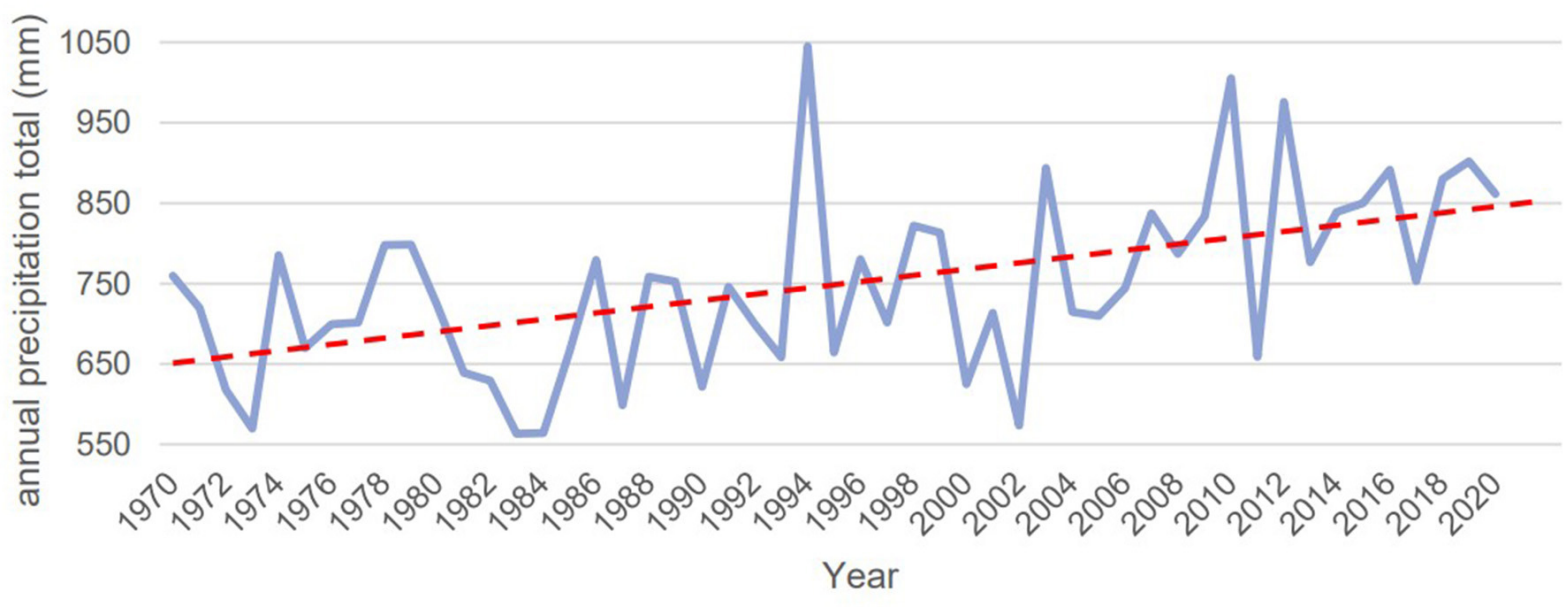
Cumulative annual rainfall from 1970 to 2020 for Nouna, Burkina Faso.

Historical data from 1970 to 2021 show an upward trend in rainfall is visible (see Figure 7). Over the last 50 years, annual rainfall has increased by 200 mm on average, rising from 650 mm to 850 mm (18).

**Figure 8:**
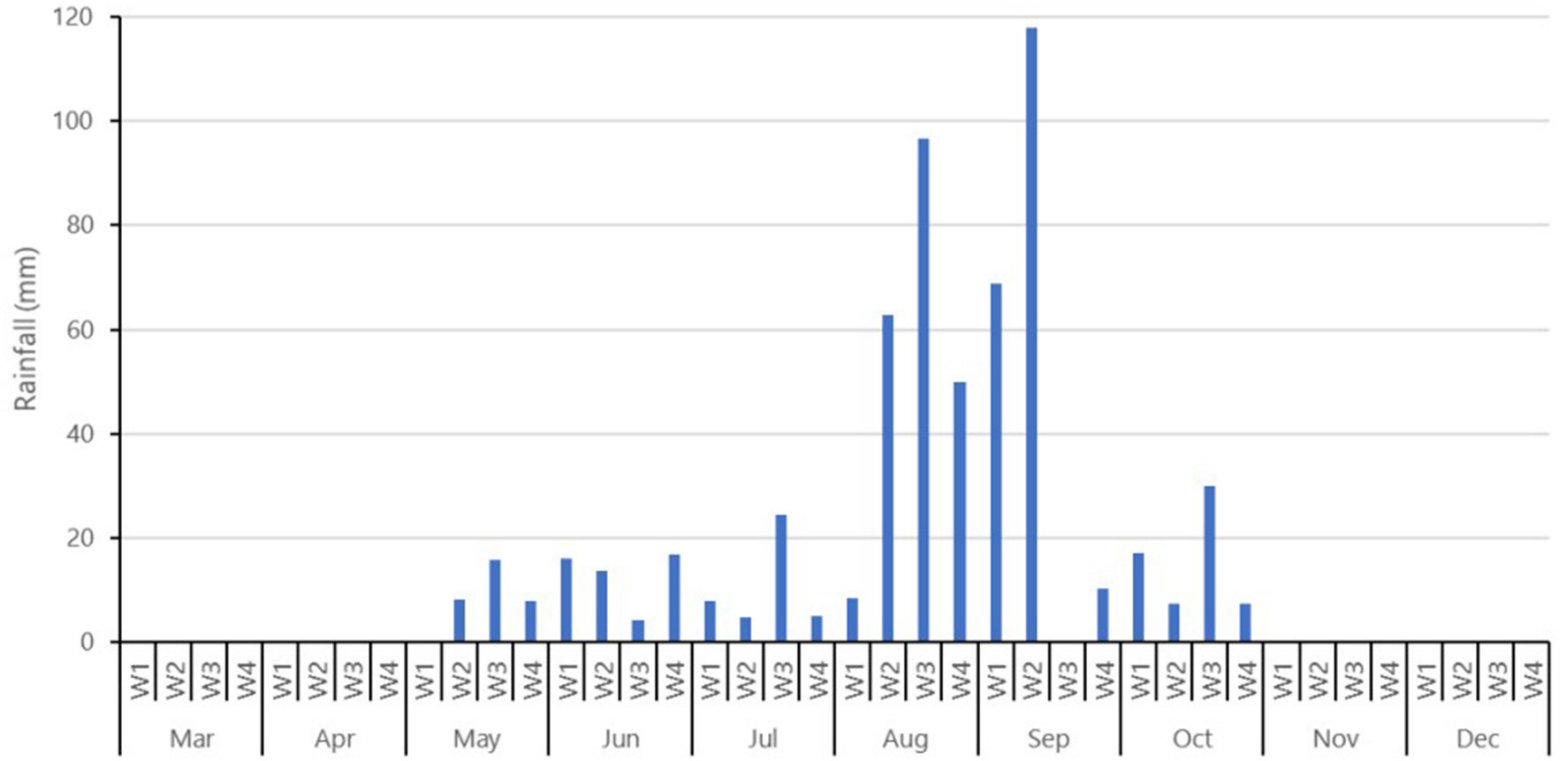
Cumulative rainfall by month for Nouna City in 2021.

Furthermore, rainfall patterns have become more erratic since 1994, characterized by steeper slopes in both directions (more extremes with regards to less and more rainfall). Consequently, not only has total annual rainfall increased, but the distribution of rainfall has also undergone changes. These factors may have contributed to more frequent flooding, as the combination of rainfall quantity and distribution can trigger flooding events. The monthly rainfall patterns provide more information regarding the frequency and intensity of heavy rainfall throughout the year (see Figure 8). In 2021, the months of July, August, and the first two weeks of September experienced the heaviest rainfall, with a measurement of 120 l/m2. Heavy rainfall is defined as 15 to 25 millimeters of rainfall within one hour or 20 to 35 millimeters of rainfall over six hours (21).

### 3.4. Example calculations of energy and economic loss due to flooding

The energy value of the agricultural cereals sorghum and millet was determined by adjusting the calculations of Belesova et al. (20). The harvest of one hectare of sorghum and millet contains an energy value of 2,043,090 and 2,181,060 kcal (22). Therefore, the energy value for the combination of crop cereal millet and cash crop cotton is 8,418,060 kcal/ha. A household of two adults and one child consumes 6,000 kcal/day (23). Accordingly, one hectare of millet could theoretically sustain a household for approximately 1 year (364 days) if just caloric needs are considered and other nutritional factors are disregarded.

Accordingly, the caloric and economic losses per hectare of cash crops were calculated. One hectare of cotton had the value of $370.83 when sold at market price (24), and sesame can be sold for $330 per hectare (25). The average monthly income for a family of four living in a rural Burkina Faso is $146 in 2020 (26). Therefore, we assume the loss of one-hectare sesame to be equivalent to two and a half months of income (see Appendix II). When considering that the average field size was 1.6 hectare, the financial loss of one field of cotton would be $593.33 correspondingly.

## 4. Discussion

This study focused on the effects of flooding in the Nouna HDSS and provided a basis for linking the components of (i) self-reported details of flooding events, (ii) satellite-based images to characterize flooding events, (iii) weather data and extreme weather events in particular heavy rainfalls, and (iv) food security in terms of an approximated energy and economic loss calculation. Within the study area in north-western Burkina Faso, we were able to gather data that characterized the magnitude and impact of flooding events. Our findings for the four components align with other relevant literature on the nexus between global warming and extreme weather events such as flooding (27). We explored four components using self-reported data collected through questionnaires, remotely sensed, satellite-based images to visually confirm flooded areas, weather station data and an exemplary calculation to quantify energy and economic loss due to flooding events.

The four-pronged approach adopted in this study—integrating participant reports, satellite imagery, weather data, and food energy loss computations—offers a comprehensive understanding of food security dynamics in rural Burkina Faso. This multidisciplinary approach allows us to assess the impact of extreme weather events on food security from multiple angles, giving a more nuanced and robust understanding of the challenges faced by subsistence farmers.

Primarily, this method enables us to identify the risks associated with climate-induced flooding more accurately and predict the potential impact on crop yields. This knowledge could be pivotal in future planning and decision-making. For instance, farmers in high-risk areas could be advised to cultivate more flood-resistant crops or employ agricultural techniques that increase resilience to flooding.

The research conducted by Witt et al. (28) provides a comprehensive analysis of flood tolerance and loss prediction for tepary beans across the United States, with a particular focus on regions such as Kansas and Oklahoma that are prone to frequent short-term flooding due to climate variability (28). This natural hazard has a significant impact on crop yield, particularly for legumes like the tepary bean that have low water tolerance. Despite these challenges, there have been no concerted breeding efforts to enhance the water resistance of this crop. The implications of this study extend beyond the immediate impact of short-term flooding on plant health. It underscores the urgent need for further research into the development of water-resistant crops, which could significantly mitigate the effects of flooding on crop yield. This is particularly relevant in the context of climate change, which is expected to exacerbate the frequency and intensity of extreme weather events such as flooding.

Waterlogging, a consequence of field flooding, is a significant yield-limiting factor for global crop production. This condition, characterized by soil saturation, restricts oxygen supply to plant roots, thereby affecting their growth and development. The extent of damage is contingent on factors such as the duration and frequency of inundation, and the developmental stage of the plant. This is corroborated by a study by Pociecha et al. (29) which examined the impact of seven-day root flooding on two differently developed field bean plants. The study found that both plants sustained damage post-flooding, with noticeable growth limitations (29). This suggests that a mere seven days of excessive water can detrimentally affect the development of field beans, a finding likely applicable to other grain legumes. Consequently, crops in regions like Burkina Faso, which are prone to similar waterlogging conditions, are likely to experience comparable challenges.

In a similar vein, the study by Müller et al. (30) provides crucial insights into the performance of sorghum cultivars under extreme weather conditions (30). The research site in N'Gakorou in the rural commune Cinzana in 2019 experienced 160% of the average precipitation since 1950, leading to significant periods of waterlogging and inundation. Despite these conditions, no severe morphological water stress signs were observed in the sorghum cultivars, underscoring the resilience of these crops under waterlogged conditions.

Enhancing water tolerance in crops is a critical factor in ensuring their survival and productivity during periods of inundation. This is evidenced by the research of Matsuura et al., which examined the waterlogging tolerance of four different types of maize. The study found significant differences among the varieties, with the maize type exhibiting larger root growth showing a greater ability to uptake water and nutrients, and maintain oxygen supply under waterlogged conditions (31). In addition to improving crop water resilience, identifying inundation zones could serve as a strategic approach to safeguarding harvests against climate change-induced flooding. These combined efforts could significantly enhance crop survival and yield under extreme weather conditions.

Moreover, the methodology employed in these studies is not confined to the specific context of Burkina Faso or the crops under investigation. With appropriate adjustments to cater to local conditions, this approach could be applied to understand the dynamics of food security in various geographic locations, with a range of different crops, and in the face of diverse natural hazards. This could provide valuable insights for the development of effective strategies to enhance food security and resilience in the face of climate change.

By employing similar methodologies, researchers could generate data crucial for informing agricultural policies and interventions aimed at improving food security and climate resilience globally. As the local population is primarily engaged in subsistence farming, their livelihoods and food security are inextricably tied to weather conditions, making them particularly vulnerable to climate-induced hazards such as flooding. Utilizing satellite imagery and weather forecasts could serve as a predictive tool to help these farmers plan better. For instance, they could cultivate water-resistant crops in areas susceptible to inundation, potentially mitigating crop failure risks. Furthermore, this research's findings may have broader applicability, extending to other African countries, such as Mali and Niger, which experience similar climatic conditions. Thus, the methodologies and insights gained from this study could inform interventions aimed at improving food security and resilience against climate change in these regions.

Given the increase in climate change-induced extreme weather events over the past decade, particularly in Africa, where droughts and flooding have become more frequent (11), and are a contributing factor to food insecurity and economic loss for subsistence farmers, such approaches as exemplified with our study are becoming increasingly important to better understand exposures and impacts to identify priorities (32).

### 4.1. The frequency of flooding and its impact on food crop harvest

The country's food security depends on the agriculture sector, especially in rural areas (12). A lower harvest means less money available to spend on health care and hygiene, which can have adverse effects on health. The results of the study manifest that 7% of all fields in the study population (59 out of 834) had lost their whole harvest due to inundation. Eight percent of all fields lost half of their harvest. In both instances, the loss of cash and crop cereal contributes to a decrease in food security.

This underlines that flooding has an impact on food security, including availability, accessibility and food stability, in this area. The most frequently consumed food, which provides 50% of peoples' daily calorie input in rural Burkina Faso, are local cereals like sorghum, millet, and maize (33). Similar effects were observed in a study from south eastern Nigeria, which linked flooding events to food security and health (34). The authors examined, if flooding affected the number of daily meals and the quality and quantity of food consumed. The national nutrition situation in Burkina Faso is overall concerning. The International Food Security Phase Classification (IPC) acute malnutrition analyses from 2022 ranked the situation in the province of Kossi and in total in 23 out of 45 provinces as “serios” or higher (“critical”). The classification is measured by how many children under five are undernourished (Integrated Food Secruity Phase Classification, 2022). Due to the high population dependent on agriculture, the country is highly vulnerable to external shocks. This can include natural disasters like droughts and flooding but also economic factors like declining prices for cotton. Both affect the country's food security; in 2019, 14.4% of the population suffered from undernutrition (35).

### 4.2. Satellite images to verify farmers' reports of flooding events

Two satellite images were used to capture the flooding in the area. In the first satellite image (20 August, 2021), the inundated areas are seen as bright blue in the color-adjusted image. The outliers of the river appear non-visible as the rain overflows the river course causing flooding. The satellite images show that large areas were underwater at that point in time. With the Sentinel-2 image we were able to visually verify the farmers' self-reported perception of the flooding and its magnitude. In the images, the red circumferences represent the agricultural fields of participants, which are partially within the inundated area. In the second satellite image (24 September, 2021), the flooding has ceased and the water has retracted to its riverbed. A change in the terrain and a greener appearance could be attributed to the favorable effects of flooding on the vegetation. However, the farmers' primary crop types, such as sorghum and millet, are cultivated traditionally because they are drought resistant. As droughts and rain shortages were the norm in Burkina Faso, the crops are drought-tolerant and cannot adapt to inundation (36).

We consider these results as proof of concept for the verification of farmers reports of flooding, which we suggest should be systematically pursued in further studies. To be able to do a systematically validation of each of the farmers flooded fields, georeferenced fields and exact dates of the reported flooding should be captured during interviews in future research. Additional research is required to establish objective analysis of satellite images and to include additional quantifications, such as harvest measurements, which was not addressed in this study. This information can be used to determine which farms have a high risk of getting flooded in the future and which crops are suitable to cultivate based on the likelihood of weather exposures. In addition, the results of a larger-scale study could help governments designate flood zones and recommend farmers to switch to water-resistant grains such as rice in order to prevent crop loss. Potentially, also the actual losses per yield of each inundated field could be measured. This would show the cumulative yearly energy loss, which in turn may be used to examine the impact of flooding on food security and other economic aspects. One example is the study by Qamer et al. (37) which utilized multi-sensor satellite data, including Sentinel-1, Sentinel-2, and Global Precipitation Measurement Mission (GPM), to assess crop production losses during floods in Pakistan. By analyzing these satellite images, the researchers were able to evaluate crop-specific losses and estimate expected production losses. This information provides a foundation for the development of evidence-based tools for loss and damage assessments in the affected areas (37).

Future research could assess the potential utility of deploying unmanned aerial vehicles (UAVs), or drones, in tandem with satellite technology, to acquire high-resolution aerial images of areas undergoing inundation events. However, the present sociopolitical instability in the study region, characterized by ongoing conflicts and terrorist activities, has introduced significant logistical impediments and safety considerations that currently preclude the use of UAVs in this research context. Notwithstanding the recognized potential of UAV-acquired data for providing granular, high-quality information, the prevailing circumstances in the study area unfortunately render the application of this technology unfeasible at present. As the optical Sentinel-2 images are highly dependent on cloudless skies, radar satellite date from Sentinel-1 for example could be a cost-free alternative or addition to drone coverage. These radar satellites can penetrate droughts and actively emit radiation, which makes them weather independent and also independent of the sunlight (38). A radar or a combined approach based on optical and radar data for surface water occurrence mapping could be used to constantly provide information about flooding events and its extents throughout the growing season (39). This analysis together with georeferenced fields and exact dates of reported flooding from interviews would enable a systematic validation approach in future research. This could not only be used as additional information to reduce the farmers vulnerability to flooding, but also to enhance crop insurances by an objective validation approach of flooding on the field level.

### 4.3. Weather data to characterize short-term and long-term trends

The characterization of weather data from 1970 to 2021 shows an upward trend for rainfall. This data indicates that rainfall has increased from 650 mm to 850 mm per year in the last 50 years. This is an average of 200 mm more rainfall in 2020 than in 1970 (18). The annual distribution of rainfall spans a broad range; in 2010, the total annual rainfall summed up to 1005.4 mm, and in 2011 decreased to 658.9 mm. This fluctuation has a large impact on droughts and flooding in the country. The insecurity of the amount of rain makes it difficult for farmers to identify which crops to sow.

The last two decades have witnessed a rise in precipitation and an increase in the frequency of flooding, according to the cumulative weather data. This aligns with other relevant literature on the nexus between climate change and extreme weather events such as flooding (27). A study by Ebi et al. (40) showed in their study, greenhouse gases, which are one driver of climate change, have an effect on the global energy balance, affecting the frequency and severity of numerous extreme weather events (40).

Additionally, projections of the cumulative annual rainfall throughout West Africa (41) and specifically for the Nouna study area (Nouna HDSS) show an increase in annual rainfall due to climate change by 2100. This will presumably worsen the situation of inundation in the country. Furthermore, Hondula et al. (42) indicate that climate change leads to severe changes in temperature and precipitation patterns in the future, liker higher variability (42).

### 4.4. Estimation of energy and economic loss due to flooding

The impact of inundation-related harvest failure on food security and undernutrition was evaluated based on a literature review (20). A study by Akukwe et al. (34) examined the relationship between flooding and food security and health by evaluating if flooding had an impact on the amount and quality of daily meals. The findings of this study are consistent with evidence reported by Akukwe et al. (34) and give comprehensible results on how harvest loss influences, directly and indirectly, food security and hence undernutrition. Calculations of energy and economic loss due to flooding could better guide interventions (adaptation, mitigation) and policies to select the right crops for different inundation risks to protect the crop and lower harvest failure due to flooding.

## 5. Limitations

One limitation of the study was no comparable flooding data from previous years were available. Therefore, it was not possible to estimate trends and put the results and findings in historic context. We verified floods visually as a constraint of the satellite-based image validation approach; furthermore, the satellite Sentinel-2 only flies over the research area every 12 days. Clouds or foggy weather limited the quality and quantity of the images. We validated floods only visually in an exemplary manner in this study as a proof of concept. The Sentinel-2 data showed its limitations due to the influence of clouds in the rainy season. A more comprehensive validation approach could be done in future research by using radar satellite data from Sentinel-1 for example. As radar satellites can penetrate clouds, the flooding extent could be monitored seamless throughout the growing period and be linked to every single field. The cumulative rainfall in Nouna in 2021 shows the highest precipitation in the second week of September, but the questionnaire did not ask for the specific timepoint of the flooding and there where to many clouds for the satellite pictures. For this reason, we can only estimate that the flooding took place between the second week of August and the third week auf September, as the average flooding of a field was 33 days long.

## 6. Conclusion

Flooding is indeed a substantial factor for crop harvest loss and thus a threat to food security, particularly for smallholder self-sufficiency farmers. This is in contrast to the ubiquitous focus on drought/mini-droughts, both in research and policy. Flooding will likely occur much more frequently in the study area and in much of sub-Saharan Africa, as a result of climate change-induced changes in weather patterns. Our estimate on the food energy loss from flooding adds further weight to the threat of “too much rain” for food security. Further to the increase of extreme precipitation, climate models project an increase in total cumulative rainfall, a combination which does not bode well for flooding risks.

More research is welcome to objectively quantify both the duration and extent (surface) of flooding. Furthermore, there is limited insights on harvest losses and the effect of climate change-related flooding on food security in vulnerable groups, such as subsistence farmers, and their relation to the four components, as described in this study. Outcomes may serve as a basis to better tailor public health interventions.

Innovative policies are necessary to safeguard farmers from harvest losses due to heavy rainfall, thus widening the current scope which is largely focused on “too little rain”, i.e., drought. This requires both the adaptation of established farming practices and the development of new ones (drone- and satellite-based surveillance of flood risks, and or example, avoiding sowing in low-lying areas), through agricultural extension workers and a reorientation of national policies.

## Data availability statement

The raw data supporting the conclusions of this article will be made available by the authors, without undue reservation.

## Ethics statement

Ethical review and approval was not required for the study on human participants in accordance with the local legislation and institutional requirements. Written informed consent from the participants was not required to participate in this study in accordance with the national legislation and the institutional requirements.

## Author contributions

CM and WO contributed to conception, design of the study, and performed the statistical analysis. WO organized the database. CM wrote the draft of the manuscript. MS took care of the Remote Sensing Part. SB and RS guided the muscribt. All authors contributed to manuscript revision, read, and approved the submitted version.

## Footnotes

^1^ www.cch-africa.de
