# Supplementary material for: The effects of climate change-induced flooding on harvest failure in Burkina Faso: case study
Source: Front Public Health. 2023 Aug 8;11:1166913. doi: 10.3389/fpubh.2023.1166913 (PMC10442567; doi:10.3389/fpubh.2023.1166913)
Supplement: Supplementary file 1 [file Table_1.DOCX]

**Supplementary Material**

**Appendix I:** Original Flooding Questionnaire in French, source: own questionnaire


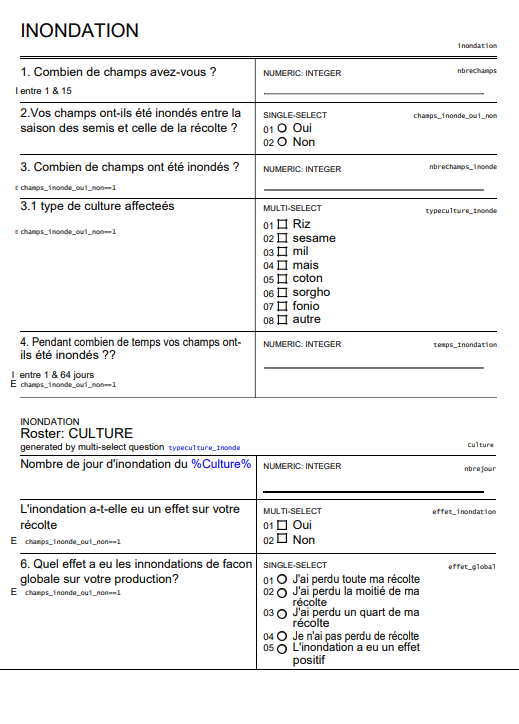


**Appendix II:** Adjusted Calculation of Energy value of crop and cash cereal produce in kilocalories/ hectare, source: (Belesova et al., 2017)

Formula:

1. Energy value of crop cereal produce in kilocalories/ hectare

E_f_ = Σ_i_ (h_i_ × c_i_)

1. Energy value of crop and cash cereals produce in kilocalories/ hectare

E_fc_ = Σ_i_ (h_i_ × c_i_) + c_i_ × Σ_c_ (h_c_ × p_c_) / p_i_

Variables:

i – food crop: millet, sorghum

c – cash crop: cotton, sesame

h – weight (kg) of the crop per ha

c – caloric value of 1 kg of the food crop

p – market price of 1 kg of food and cash crop

| Crop Type | Crop Outcome (kg / ha) | Market price ($ / kg) | Energy value for food crop (kcal/kg) |
| --- | --- | --- | --- |
| Sorghum | 621 | 0.084 | 3290 |
| Millet | 577 | 0.2 | 3780 |
| Sesame | 378.4 | 0.98 | / |
| Cotton | 750 | 0,44 | / |

Table: Crop type outcome in kg/ha, market price and energy value, source: own table, data: (Sogoba et al., 2020), (Dossa et al., 2017), (M. Sabesh & A. H. Prakash, 2018), (Selina Wamucii, 2022), (sofitex, 2022) (NutritionValue.org, 2022)

Calculation:

1. Energy value of sorghum produce in kilocalories/ hectare

E_f_ = Σ_i_ (621kg/ha × 3290kcal/kg) = 2,043,090 kcal/ha

1. Energy value of Millet produce in kilocalories/ hectare

E_f_ = Σ_i_ (577kg/ha × 3780kcal/kg) = 2,181,060 kcal/ha

1. Energy value of millet and cotton produce in kilocalories/ hectare

E_fc_ = Σ_i_ (577kg/ha × 3780kcal/kg) + 3780kcal/kg × Σ_c_ (750kg/ha × 0,44$/kg) / 0.2$/kg = 8,418,060 kcal/ha

1. Energy value of sorghum and sesame produce in kilocalories/ hectare

E_fc_ = Σ_i_ (621kg/ha × 3290kcal/kg) + 3290kcal/kg × Σ_c_ (378.4kg/ha × 0,98$/kg) / 0.084$/kg = 16,567,343.22 kcal/ha

1. Energy loss per Family of three per day / year

8,418,060 kcal/ha / 6000 kcal/day = 1403 days / 365 = 3,84 years

1. Economic loss in U.S. dollar / hectare

Cotton: 378.4kg/ha x 0.98$/kg = 370,83 $/ha

Sesame: 750 kg/ha x 0,44$/kg = 330 $/ha

1. Financial loss of one hectare of sesame(330$) compared to a monthly income(146$)

Calculation: 330$/146$= 2.3 month

**Appendix III:** Field size calculation

| Variable | Obs | Mean | Std. Dev. | Min | Max |
| --- | --- | --- | --- | --- | --- |
|  |  |  |  |  |  |
| Field_size | 825 | 1.633333 | 1.608324 | 0,77 | 3,74 |
